# A typical presentation of AMSAN variant of guillain-barré syndrome in an elderly female with multisystem involvement: A case report

**DOI:** 10.6026/973206300212181

**Published:** 2025-07-31

**Authors:** Pratap Singh Sekhon, Manish Pendse, Neha Momale

**Affiliations:** 1 Department of General Medicine, D Y Patil University, School of Medicine, Nerul, Navi Mumbai, India

**Keywords:** Syndrome, Acute Motor and Sensory Axonal Neuropathy (AMSAN), Prognosis, Cranial Nerve, MRI, EEG, Neurophysiological

## Abstract

Guillain-Barré Syndrome (GBS) is an acute immune-mediated polyradiculoneuropathy, with the AMSAN (Acute Motor and Sensory Axonal Neuropathy) variant representing a severe subtype characterized by rapid progression and poor prognosis. This report describes a 68-year-old woman presenting with progressive quadriparesis, cranial nerve involvement, and bowel and respiratory complications, ultimately diagnosed as AMSAN based on nerve conduction studies and cerebrospinal fluid analysis. MRI and EEG findings contributed to the diagnostic complexity due to age-related degenerative and vascular changes. Despite early IVIg administration, the patient required intensive care, emphasizing the need for prompt diagnosis and multidisciplinary management. AMSAN in elderly patients demands high clinical vigilance and early neurophysiological evaluation to initiate timely immunotherapy and improve outcomes.

## Background:

Guillain-Barré Syndrome (GBS) is a short-lived, immune-mediated polyradiculoneuropathy that constitutes a neurologic emergency with rapid diagnosis and management necessary [1]. Characteristically involving weakness, often descending progressive symmetry in limbs, and variable sensory and autonomic disturbances along with areflexia, GBS classically arises subsequent to an antecedent infection with subsequent worsening spanning several days or weeks [2]. Though the most frequent of cases are demyelinating in character typically referred to as Acute Inflammatory Demyelinating Polyneuropathy (AIDP) there also exist less prevalent axonal types with more serious pathology and that frequently have poorer prognosis [3]. The Acute Motor and Sensory Axonal Neuropathy (AMSAN) form is an uncommon and severe form of GBS. Initially reported in the mid-1990s in Asian patients, AMSAN is characterized by diffuse axonal degeneration involving both motor and sensory nerves [4]. Its pathophysiology is different from that of AIDP, with direct axonal injury rather than segmental demyelination, frequently leading to more severe weakness, sensory impairment, and incomplete or delayed recovery [5]. These patients usually have little or no demyelination on nerve conduction studies, but rather low amplitude or absent motor and sensory action potentials, which are indicative of axonal loss. Histologically, AMSAN is linked with macrophage-mediated axonal degeneration and ganglioside antibody positivity, specifically anti-GD1a and anti-GM1 [6]. The clinical and electrophysiological diagnosis of AMSAN is the mainstay. But in older patients, comorbid conditions such as degenerative spinal disease, cerebrovascular alterations, and organ system dysfunction due to age conceal or simulate the presentation, making the diagnosis elusive on time [7]. The additional diagnostic pitfalls include the presence of AMSAN in combination with features that are atypical, including cranial nerve palsy, weakness of respiratory muscles, bowel or bladder dysfunction, or central nervous system imaging findings [8]. These presentations tend to result in lengthy investigations and misdiagnoses, which delay the onset of definitive immunotherapy like intravenous immunoglobulin (IVIg) or plasmapheresis [9]. Prognosis of AMSAN is usually guarded. In contrast to AIDP, which tends to have excellent response to IVIg and early improvement in function, AMSAN can lead to long-lasting or incomplete recovery from the injury [10]. Recovery can take months to years, and some are left severely disabled irrespective of the best management. Also, elderly patients with AMSAN tend to have poorer physiological reserves and are more likely to develop complications such as respiratory failure, sepsis, autonomic instability, and bowel dysfunction [11]. This report discusses a 68-year-old woman who came with rapidly progressive flaccid quadriparesis, eventually diagnosed as AMSAN variant of GBS on the basis of neurophysiology and clinical examination [12]. The case is significant in its multifaceted course, with multisystem complications and intensive care management. It underscores the diagnostic challenge presented by AMSAN in the elderly, the essence of nerve conduction studies in distinguishing between axonal and demyelinating disease, and the necessity of a team approach to optimal care [13]. Therefore, it is of interest to report this case to contribute to the growing literature on atypical GBS presentations and highlight the importance of early recognition and multidisciplinary management in high-risk elderly patients.

## Case presentation:

A 68-year-old lady farmer from Satara, Maharashtra, reported to the emergency department on August 12, 2024, with pain and gradually increasing weakness in both the upper and lower limbs for five days. Pain was initially presented as deep, aching, and symmetrical involving mainly cervical and lumbar regions. During the next four days, the patient had progressive weakness in all four limbs, without any accompanying trauma, onset fever, or systemic illness. She also noted the onset of hoarseness and dysphonation one day before presentation, after the placement of a right-sided nasogastric tube. No history of facial deviation, seizures, loss of consciousness, urinary or fecal incontinence, or antecedent diarrheal or respiratory illness. Notably, the patient had a similar episode of limb pain a few days before this current admission for which she had sought care at a local hospital. At that time, she experienced partial symptomatic relief with analgesics, but the condition recurred and progressed. Her past medical and surgical history was unremarkable. She was not known to have diabetes, hypertension, or other chronic systemic illnesses. There was no significant family history of neurological or autoimmune disease.

On neurological examination at presentation, the patient was conscious and oriented. Her higher mental functions were intact. Cranial nerve examination revealed a weak gag reflex and reduced phonation. Motor examination demonstrated hypotonia in all four limbs. Power was graded as 2/5 in both upper limbs and 1/5 in both lower limbs (Medical Research Council scale). Deep tendon reflexes, including biceps, triceps, knee, and ankle jerks, were absent bilaterally. Plantar responses were asymmetric: the right side was absent, and the left was extensor. Sensory examination was largely intact. Meningeal signs were absent, and cerebellar assessment could not be performed due to the profound weakness. No autonomic disturbances were initially noted. Laboratory investigations revealed a mildly elevated creatine phosphokinase-MB (CPK-MB) of 46.76 U/L and markedly reduced serum cholinesterase levels (7148.1 U/L, later declining to 2838 U/L), suggesting possible toxic or inflammatory neuromuscular involvement. MRI of the whole spine on August 10 showed widespread degenerative changes, including Grade I-II anterolisthesis of L5 over S1 and multilevel disc bulges in the cervical and lumbosacral spine, many of which encroached on the anterior thecal sac and neural foramina. However, these findings were not sufficient to explain the rapidly progressing flaccid quadriparesis. NCV (Nerve Conduction Velocity) study performed on August 14 was strongly suggestive of a sensory-motor axonal neuropathy, affecting the lower limbs more severely than the upper limbs, with features of secondary demyelination - findings consistent with the AMSAN variant of GBS. CSF analysis revealed mildly elevated proteins (44 mg/dL) and elevated white cell count (20 cells/cmm with 90% neutrophils), though albuminocytologic dissociation was not prominent at this stage. Initial treatment was started with intravenous immunoglobulin (IVIg) at 400 mg/kg/day for 5 days, continued for 2 more days based on clinical monitoring. Despite therapy, there was only minimal neurological improvement after two weeks, with repeat CNS examination on August 28 showing a marginal increase in upper limb power (3/5), while lower limb power remained at 1/5. Gag reflex remained sluggish, and plantar responses were bilaterally absent.

MRI brain perform on August 10 and again with contrast on August 20 reveals old lacunar infarcts, gliotic changes, and diffuse cerebral and cerebellar atrophy, in addition to a possible developmental venous anomaly and questionable hemipontine lesion, likely of vascular or infective etiology. EEG showed right frontotemporal and left centroparietal slowing with occasional sharp waves. These findings, while nonspecific, raised the concern of pre-existing or evolving cerebrovascular compromise. The patient's condition further deteriorated with the development of gastrointestinal symptoms including constipation, abdominal bloating, and fever. CECT abdomen and pelvis on August 24 revealed long segment dilatation and thickening of the large bowel, particularly in the distal sigmoid colon, with fecal loading and signs of inflammation. Colonoscopy performed on September 9 revealed proliferative thickening in the descending and sigmoid colon, for which histopathological correlation was advised. During hospitalization, the patient also developed fever (up to 102°F), loose stools, and increased respiratory rate (RR 44/min), necessitating transfer to the medical ICU (MICU). Repeat neurological assessment revealed persistence of lower limb power at 0/5 in most muscle groups, with 1/5 at bilateral ankles. Upper limb strength remained limited to the elbows (2/5), while reflexes remained absent in all limbs. Repeat NCV confirmed the evolving nature of axonal sensory-motor neuropathy. Further investigations showed elevated inflammatory markers (CRP increased from 11.67 to 29.6 mg/L), while serum electrolytes and renal parameters remained within acceptable ranges. CSF cytology remained acellular, with mildly elevated protein and glucose. Medications administered included broad-spectrum antibiotics (IV Magnex Forte, IV Metronidazole), neuroprotective agents (Levipill, Neurewire), antihypertensives (Ivabradine, Cilnidipine-Telmisartan combination), potassium correction, and supportive measures for gut and fluid balance. Despite aggressive multidisciplinary care involving neurology, internal medicine, orthopedics, and neurosurgery consultations, the patient exhibited only partial clinical improvement and remained dependent for basic care at the time of report compilation.

## Discussion:

Guillain-Barré Syndrome (GBS) is a heterogeneous group of immune-mediated neuropathies. While Acute Inflammatory Demyelinating Polyneuropathy (AIDP) is the most common variant globally, axonal variants such as Acute Motor Axonal Neuropathy (AMAN) and Acute Motor and Sensory Axonal Neuropathy (AMSAN) are less frequent but often more severe in presentation and outcome [14]. The AMSAN variant, first identified in Northern China and later reported in Japan and South America, is characterized by rapid onset of distal and proximal weakness, profound sensory loss, and absent reflexes due to severe axonal degeneration of both motor and sensory nerves [15]. In this case, a 68-year-old woman developed classical signs of GBS, including bilateral limb weakness, loss of reflexes, and cranial nerve involvement, specifically reduced phonation and a weak gag reflex. Neurophysiological testing confirmed axonal sensory-motor neuropathy, consistent with AMSAN, and CSF showed mild protein elevation with neutrophilic pleocytosis. While albuminocytologic dissociation a hallmark of GBS is often absent early in axonal variants, the evolving CSF findings in this case support the inflammatory nature of the illness [16]. This case is particularly unique due to the multifaceted diagnostic and management complexities encountered in the elderly. Geriatric patients pose a distinct challenge, as degenerative spine disease may mimic lower motor neuron signs, making it difficult to distinguish early between compressive myelopathy and peripheral neuropathy [17]. Additionally, cerebrovascular changes such as lacunar infarcts and gliosis, observed in the patient's MRI, can further obscure the neuromotor manifestations attributable to Guillain-Barré Syndrome (GBS) [18]. Compounds these issues, reduced physiological reserves in older adults increase vulnerability to rapid systemic decompensation, as evidenced by the patient's gastrointestinal and respiratory complications. These overlapping factors necessitate a comprehensive, multidisciplinary approach for accurate diagnosis and effective management [19].

One notable feature in this patient was the development of bowel symptoms with imaging evidence of distal colonic dilatation and inflammation, possibly representing autonomic involvement of the gastrointestinal tract a recognized but uncommon manifestation of GBS [20]. Similarly, the respiratory distress requiring MICU transfer reflects either autonomic instability, neuromuscular weakness of respiratory muscles, or underlying infection. Both are well-documented complications in severe GBS and particularly in AMSAN, which has a higher tendency to cause life-threatening progression due to rapid axonal injury [21]. The case also emphasizes the diagnostic role of neurophysiological studies, which were crucial in establishing the diagnosis when MRI findings were nonspecific and brain imaging suggested concurrent unrelated pathology. The NCV confirmed the presence of low amplitude motor and sensory potentials, consistent with axonal loss and distinguishing the presentation from demyelinating forms [22]. Treatment with IVIg at standard dosing (400 mg/kg/day for 5 days) remains the cornerstone of therapy, with plasmapheresis being an alternative. The limited neurological improvement in this patient, particularly in the lower limbs, is aligned with previous literature indicating that AMSAN often has a slower and incomplete recovery trajectory [23]. Unlike AIDP, where remyelination allows for relatively quick restoration of function, AMSAN involves primary axonal degeneration, which necessitates axonal regrowth a process that is typically slow and sometimes irreversible [24]. Electrophysiological recovery in AMSAN often lags behind clinical improvement and may continue to evolve for months. In this patient, follow-up NCV confirmed the persistent evolving nature of the axonal insult, reinforcing the need for long-term rehabilitation planning and monitoring [25].

## Conclusion:

The diagnostic complexity and guarded prognosis of the AMSAN variant of Guillain-Barré Syndrome in elderly patients with overlap comorbidities. Early neurophysiological testing and a multidisciplinary approach were essential for accurate diagnosis and management. Despite prompt IVIg therapy, the patient's limited recovery highlights the aggressive nature of axonal variants and the critical need for vigilant monitoring and supportive care.
